# Factors associated with choice of approach for Group B streptococcus screening

**DOI:** 10.1186/s13584-016-0103-6

**Published:** 2016-11-15

**Authors:** H. Sefty, A. Klivitsky, M. Bromberg, R. Dichtiar, M. Ben Ami, T. Shohat, A. Glatman-Freedman, For the Israel Obstetric Survey Group (IOSG), E. Anteby, R. Auslander, I. Ben Schachar, J. Borenstein, B. Chayen, L. Harel, N. Farah, A. Fishman, A. Golan, O. Gonen, Z. Hagay, M. Hakim, M. Hallak, A. Herman, N. Idays, J. Itskovitz, R. Kasrawi, A. E. Kharouf, N. Laufer, J. Lessing, Y. Mamet, M. Mazor, T. Nseir, R. N. Pollack, A. Samueloff, E. Schiff, E. Shalev, A. Wiznitzer, S. Yagel, A. Zabari, H. Amsalem, M. Bardicef, N. Braverman, N. Cohen, A. Cohen-Arazi, A. Eliasy, R. Gur, D. Haim, I. Hendler, D. Ismail, A. Israeli, I. Jahshan, M. Kehat, A. Kharouf, T. Khodak-Be’eri, A. Kopitman, R. Levy, Z. Luria, D. Mankuta, S. Nahir-Biderman, K. Ovadia, Y. Perlitz, O. Reichman, O. Sadan, R. Salim, P. Schwed, Y. Shaham, S. Shahar, E. Vaisbuch, A. Weissman, M. Yosef-Barin, Y. Zakharian, O. Zinger, N. Ziv

**Affiliations:** 1 Israel Center for Disease Control, Ministry of Health, Tel Hashomer, Israel; 2 Pediatric Infectious Disease unit, E.Wolfson Medical Center, Holon, Israel; 3 Department of Obstetrics and Gynecology, Poriya Medical Center, Tiberias, Israel; 4 School of Medicine, Bar Ilan University, Ramat Gan, Israel; 5 School of Public Health, Sackler School of Medicine, Tel Aviv University, Tel Aviv, Israel; 6 Departments of Pediatrics and Family and Community Medicine, New York Medical College, Valhalla, NY USA; 7 The Israel Center for Disease Control, Gertner Institute, Chaim Sheba Medical Center, Tel Hashomer, 52621 Israel

**Keywords:** Group B streptococcus, Screening, GBS carrier

## Abstract

**Background:**

The crude rate of early-onset Group B streptococcus disease (EOGBS) in Israel has been consistently under 0.5 for 1000 live births for the past 8 years. The Israeli Ministry of Health has adapted the risk factor based approach for preventing EOGBS and universal bacteriological screening for GBS is not recommended. In spite of this policy, there are indications that many pregnant women in Israel undergo bacteriological screening for GBS.

The objective of this study is to assess the rate and characteristics of pregnant women who undergo screening for group B streptococcus (GBS) colonization in Israel.

**Methods:**

Survey of expectant mothers who came to give birth in 29 delivery rooms throughout Israel during the month of July 2012 regarding GBS screening practice and demographics.

**Results:**

A total of 2968 pregnant women participated in the assessment. Among them, 935 women (31.5 %) had been tested for GBS colonization. About 90 % of those women had no risk factors, only 542 women (60 %) underwent testing during the recommended gestational timing (35–37 weeks) and 23 % of the tested women reported being GBS carriers.

GBS screening as part of the routine pregnancy follow- up was associated with: residence district, intermediate or high socioeconomic rank, being a member of certain health maintenance organization and being Jewish.

Characteristics found to be significantly associated with being a GBS carrier were: low socioeconomic rank, and having a risk factor for GBS infection.

**Conclusions:**

A substantial number of pregnant women in Israel undergo screening for GBS colonization despite the national policy against universal screening. While GBS colonization was more prevalent in women of lower socioeconomic status, screening is done more often in those of higher socioeconomic status, suggesting unnecessary monetary expenses.

## Background

Group B Streptococcus (GBS) is a leading cause of life-threatening infection in newborns causing sepsis, pneumonia and meningitis [1]. Early onset GBS (EOGBS) occurs within the first week of life (0–6 days) [2]. Intra-partum antibiotic prophylaxis (IAP) has been proven to lower the incidence of EOGBS [2, 3]. Screening for women requiring IAP has been done using one of two approaches: culture-based universal screening which should be done between weeks 35 and 37 of pregnancy and risk-based approach in which women receive IAP based on the presence of risk factors [1, 4]. Universal Screening policy is practiced in the United States and Canada [1–3, 5] and it is also recommended with some modifications in many European countries and in Japan [6], (http://www.groupbstrepinternational.org/what-is-group-b-strep/early-onset-gbs-disease/), [2]. A risk-based approach is recommended in Denmark, Israel, the Netherlands, New Zealand and the United Kingdom [1, 2]. Prior to the introduction of preventive measures, the incidence of EOGBS ranged between 0.5 to more than 4 per 1000 live births, and the rates varied substantially among various geographical regions [2].

Israel has adopted the risk-based approach in which pregnant women are not routinely screened for GBS carriage [7], but rather receive IAP if they have at least one of the following risk factors: labor before week 37, prolonged (above 18 h) rupture of membranes, fever above 38 °C during labor, a previous infant with GBS disease, and GBS bacteriuria at any stage of pregnancy.

Israel has been monitoring this policy since 2006 through active yearly national surveillance of all newborns with EOGBS. The crude multi-year incidence from 2010 to 2014 was 0.26 per 1000 live births. Among infants whose mothers had risk factors, the incidence was 0.50 per 1000 live births, while the multi-year incidence among infants whose mothers had no risk factors was 0.20 per 1000 live births [8].

Although there is no universal screening in Israel, many women are tested for GBS carriage during pregnancy (personal communication). As a result, the Israeli Society of Obstetrics and Gynecology position is that IAP should be administered both to women who have at least one risk factor and to women who have been tested for GBS carriage close to labor and found to be colonized [9].

The objectives of our study were to assess the rate of women who undergo the testing for GBS carriage in Israel, their demographic characteristics and the reasons for performing the test.

## Methods

### Study subjects

Pregnant women who came to give birth in delivery rooms throughout Israel from July 1, 2012 to July 31, 2012 were included in the survey. Women whose referral to the delivery room did not result in labor and non-residents were excluded from the study.

### Survey

The survey was administered during weekday morning shifts throughout the month of July 2012 (a total of 23 days) in 29 delivery rooms (out of total 30) throughout Israel. A nurse (or any other previously appointed personnel member) filled out a short questionnaire for each woman who came to give birth. The questionnaire included information on last menstrual date, whether a GBS testing was done, the timing of the test, the result of the test, and the reason for performing it. Demographic information included woman’s age, country of origin, Health Ministry residence district, population group, and health insurance membership. In cases where the GBS test was positive, women were asked to show documentation of the result.

### Determination of socioeconomic status

Socioeconomic status was based on women’s place of residence, using the Israel Central Bureau of Statistics (CBS) definition [10]. The CBS ranks each municipality into 1 of 10 socioeconomic clusters, 1 being the lowest and 10 being the highest. Each woman was assigned one of 3 socioeconomic ranks based on the socioeconomic cluster of her place of residence: low (for clusters 1–3), intermediate (clusters 4–6), and high (clusters 7–10).

### Statistical analysis

Comparison of continuous variables was performed using the Students’ *T*-test (assuming normal distribution), and comparison of categorical variables was done using the Chi square test. Multivariate analysis was performed using logistic regression analysis. A *p* value <0.05 was considered statistically significant. The statistical analysis was carried out using the SAS software (version 9.1.3).

### Ethical consideration

Performance of the survey was approved by the legal Council of the Israel Ministry of Health, in accordance with the public health act enacted in Israel. Under this act the Israel Ministry of Health can perform surveillance and monitoring of the performance of health policies and directives. As such it does not require any special consent beyond the expectant mother general agreement to receive accepted appropriate labor and delivery room treatment.

## Results

### Rate of GBS testing

**Fig. 1:**
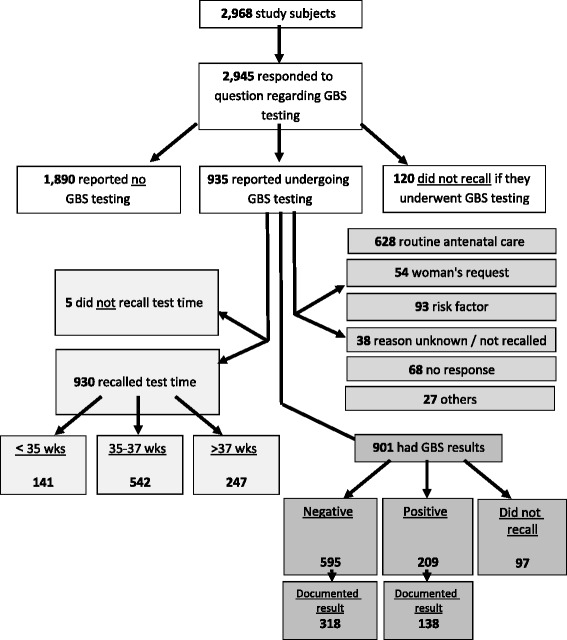
Flow chart of parturient women participating in the survey

A total of 3015 pregnant women came to give birth in 29 hospitals throughout Israel, during the time of the survey. Of those, 47 were excluded as they were not Israeli residents. Of the 2968 women who participated in the survey, 2945 (99.2 %) responded to the question on GBS testing. Of those, 935 (31.7 %) reported having undergone the test, 1890 (64.3 %) did not and 120 (4 %) could not recall if they had undergone GBS testing. Fig. 1 presents a flow chart that outlines the distribution of study participants.

### Timing of GBS testing

Of the 935 women who reported undergoing GBS testing, 930 (99.5 %) were able to recall the timing of the test (Fig. 1). Of those, 542 (58.2 %) underwent the test between 35 and 37 weeks, 141 (15.2 %) had it before week 35, and 247 (26.5 %) after week 37.

### Reasons for GBS testing

Of the 935 women who reported having undergone the GBS screening, 867 (92.7 %) responded to the question regarding the reasons for being tested. A total of 628 women (72.4 %) underwent the test as a part of the routine pre-natal care and additional 54 women (6 %) as a result of their request (Fig. 1, Table 1). Only 120 women (14 %) reported undergoing GBS testing due to a medical reason: previous GBS infection in a newborn infant, Rupture of membranes before week 37, Contractions with marked cervix changes before week 37, GBS bacteriuria in the current pregnancy or GBS carrier in previous pregnancy. Other medical reasons given for undergoing GBS testing were: urinary tract infection (UTI), recurrent UTI, UTI symptoms, vaginal discharge/odor/infection, intrauterine growth retardation (IUGR) and hospitalization during pregnancy (unspecified reason) (Fig. 1). Table 1 presents the specific reasons reported for undergoing GBS testing. 77.2 % of women who underwent GBS testing between 35 and 37 weeks reported that the main reason for undergoing GBS testing was as part of the routine pre-natal care.

**Table 1:** Reasons reported for GBS testing during pregnancy

Timing	Any		<35 weeks		35–37 weeks^a^		>37 weeks	
Reason	*n*	(%)	*n*	(%)	*n*	(%)	*n*	(%)
Routine pre-natal care	628	72.4	65	48.5	396	77.2	165	75.7
Woman’s request	54	6.2	3	2.3	33	6.4	18	8.3
Previous GBS infection in a newborn infant	23	2.7	1	0.8	17	3.3	5	2.3
ROM before week 37	7	0.8	3	2.2	4	0.8	-	-
Contractions with marked cervix changes before week 37	38	4.4	26	19.4	10	2.0	2	0.9
GBS bacteriuria in the current pregnancy	27	3.1	13	9.7	8	1.6	6	2.7
GBS carrier in previous pregnancy	27	3.1	3	2.2	16	3.1	8	3.7
Other	25	2.9	11	8.2	10	1.9	4	1.8
Reason unknown/not recalled	38	4.4	9	6.7	19	3.7	10	4.6
Total No. of women who responded to question	867	100	134	100	513	100	218	100

^a^For two women the gestational age was known, however no reason was recorded on questionnaire. They were categorized as missing information and are not included in the table

Women who underwent GBS testing before week 35 or after week 37 (Table 1) also reported that the main reason for undergoing the test was as part of the routine pre-natal care. This reason was reported by 48.5 % of the women who underwent the test prior to week 35, and 75.7 % of women who underwent the test after week 37 (Table 1).

We compared women who underwent GBS testing as part of routine prenatal care to women who underwent the test for other reasons with respect to the various gestational week category (<35, 35–37 and >37) in which the test was performed. Women who underwent GBS testing between 35 and 37 weeks of gestation and after week 37 reported that routine pre-natal care was the main reason for undergoing the test significantly more often than women who underwent GBS testing before week 35 (*p* < 0.0001). No statistically significant differences were observed when comparing women who underwent the test during weeks 35 to 37 with women who underwent the test later than week 37 of pregnancy. Similar results were obtained for the comparison between women who underwent GBS testing as part of routine prenatal care to those who underwent the test for medical reasons.

### GBS test results

GBS screening test results were reported by 901 women (96.3 %). A total of 209 (23.2 %) of the women reported a positive culture for GBS, 595 (66.0 %) were negative and 97 (10.8 %) could not recall the results (Fig. 1).

A total of 804 women provided a response both regarding the reason for having had the test and its result. For 131 women, information on the reason for GBS screening and/or its results was not available. Of the 935 women who reported undergoing the GBS test, 473 (50.6 %) presented a documented result of the test. A total of 138 (66 %) of the 209 GBS carriers presented a documentation of a positive test and 318 (53.4 %) of the 595 non-GBS carriers presented a documentation of a negative test. For 17 women who reported undergoing the GBS test and presented a documented result, the result was not recorded.

Women who were GBS-tested for medical reasons were significantly more often positive for GBS compared with those who had it as part of the routine prenatal care (43.2 % vs 21.9 %, *p* < 0.0001, odds ratio 2.71) (Table 2).

**Table 2:** Distribution of GBS screening results with respect to the presence of a risk factor

Risk Factor	Positive *n* (%)	Negative *n* (%)	Unknown *n* (%)	Total *n*
Yes	38 (43.2)	43 (48.9)	7 (7.9)	88
No	157 (21.9)	494 (69.0)	65 (9.1)	716
Total	195	537	72	804

### Demographic characteristics

The characteristics of the women who performed the test were compared with those who did not. The univariate analysis showed statistically significant differences in Health Ministry residence district, country of origin, socioeconomic rank, HMO membership and population group between women who underwent GBS testing and those who did not. No difference was found in the mean age of the women who took the test and those who did not. In the multivariate analysis (Table 3), statistically significant differences were found for all variables, except and country of origin (Table 3). Specifically, characteristics that were significantly associated with GBS testing were: belonging to the Jerusalem district, belonging to intermediate and high socioeconomic rank, being a member of certain HMOs and being Jewish (Table 3). When the analysis was restricted to those women who had documentation of their GBS testing, belonging to the Jerusalem district, belonging to intermediate and high socioeconomic rank, being a member of certain HMOs and being Jewish were associated with women who underwent GBS testing in a multivariate analysis. Additionally, when the analysis was restricted to those women who performed the test because of routine pre-natal care, differences in Health Ministry residence district were associated with women who underwent GBS testing because of routine pre-natal care, in a multivariate analysis.

**Table 3:** Relationship between GBS testing status and demographic characteristics using univariate and multivariate analysis

Characteristic		GBS testing *n* (%) *n* = 935	No GBS testing *n* (%) *n* = 1,890	Univariate Analysis			Multivariate Analysis		
				Odds Ratio	95 % CI	*p* value	Odds Ratio	95 % CI	*p* value
Average age (years)		29.5	29.3	-		0.61			
Residency district *n* (%)	Jerusalem	219 (23.4)	282 (14.9)	1		Ref	1		Ref
	North	76 (8.1)	398 (21.1)	0.2	0.2–0.3	<0.0001	0.3	0.2–0.4	<0.001
	Haifa	60 (6.4)	183 (9.7)	0.4	0.3–0.6	<0.0001	0.2	0.1–0.4	<0.001
	Center	169 (18.1)	455 (24.1)	0.5	0.4–0.6	<0.0001	0.2	0.2–0.4	<0.001
	Tel Aviv	125 (13.4)	254 (13.4)	0.6	0.5–0.8	<0.01	0.3	0.2–0.4	<0.001
	South	187 (20.0)	239 (12.6)	1.0	0.8–1.3	0.9	0.9	0.7–1.2	0.5
	Judea & Samaria	99 (10.6)	79 (4.2)	1.6	1.1–2.3	<0.01	0.9	0.7–1.4	0.8
Country of origin *n* (%)	Israel	754 (81.6)	1511 (84.0)	1		Ref	1		Ref
	Asia-Africa	19 (2.1)	56 (3.1)	0.7	0.4–1.2	0.2	0.7	0.4–1.2	0.2
	America-Europe	151 (16.3)	232 (12.9)	1.3	1.1–1.7	<0.01	0.9	0.7	0.5
Socioeconomic rank *n* (%)	Low	63 (7.0)	288 (16.7)	1		Ref	1		Ref
	Intermediate	620 (69.0)	1073 (62.2)	2.6	2.1–3.3	<0.0001	1.9	1.4–2.6	<0.001
	High	216 (24.0)	365 (21.1)	2.7	2.0–3.5	<0.0001	3.2	2.1–4.7	<0.001
Health Maintenance Organization *n* (%)	Clalit Health service	402 (43.6)	1089 (58.8)	1		Ref	1		Ref
	Maccabi Healthcare services	229 (24.8)	361 (19.5)	1.7	1.4–2.1	<0.0001	1.5	1.2–1.8	<0.001
	Le’umit	93 (10.1)	164 (8.8)	1.5	1.1–2.0	<0.01	1.1	0.8–1.5	0.3
	Me’uhedet	198 (21.5)	238 (12.8)	2.2	1.8–2.8	<0.0001	1.6	1.2–2.0	<0.001
Religion *n* (%)	Arab	89 (9.5)	570 (30.1)	1		<0.0001	1		<0.001
	Jewish & others	846 (90.5)	1320 (69.8)	4.1	3.2–5.2		2.6	1.9–3.5	

We also evaluated the differences in demographic characteristics between women who reported being GBS carriers and those who reported being a non-carriers (Table 4). Slightly younger age (1.2 years difference in mean age), low socioeconomic status, having a medical reason for GBS testing and Arab population group, were significantly associated with GBS positivity in a univariate analysis. In a multivariate analysis the characteristics that were most significantly associated with a GBS carrier state were: low socioeconomic status and having a medical reason for GBS testing (Table 4). Belonging to a certain HMO was also associated with a GBS carrier state. When the analysis was restricted to those women who had documentation of their GBS status, low socioeconomic status and having a Medical reason for GBS testing were associated with GBS carrier state in a multivariate analysis.

**Table 4:** Demographic characteristics of GBS carriers and non-carriers using univariate and multivariate analysis

Characteristic		Carriers *n* (%) *n* = 209	Non-carriers *n* (%) *n* = 595	Univariate Analysis			Multivariate Analysis		
				Odds Ratio	95 % CI	*p* value	Odds Ratio	95 % CI	*p* value
Average age (years)		28.6	29.9	-		0.002			
Residency district *n* (%)	Jerusalem	53 (25.4)	132 (22.2)	1		Ref.	1		
	North	18 (8.6)	40 (6.7)	1.1	0.6–2.1	0.7	0.7	0.3–1.6	0.4
	Haifa	13 (6.2)	43 (7.2)	0.7	0.3–1.5	0.4	0.9	0.4–2.1	0.9
	Center	27 (12.9)	121 (20.3)	0.5	0.3–0.9	0.0	0.7	0.3–1.2	0.2
	Tel Aviv	30 (14.3)	86 (14.4)	0.9	0.5–1.4	0.6	1.2	0.6–2.4	0.6
	South	44 (21.0)	108 (18.1)	1.0	0.6–1.6	0.9	0.9	0.5–1.6	0.8
	Judea & Samaria	24 (11.5)	65 (10.9)	0.9	0.5–1.6	0.7	0.9	0.5–1.8	0.9
Country of origin *n* (%)	Israel	162 (80.6)	485 (81.6)	1		Ref.	1		
	Asia-Africa	5 (2.5)	11 (1.85)	1.3	0.4–3.8	0.6	1.9	0.6–6.2	0.2
	America-Europe	34 (16.9)	98 (16.5)	0.9	0.6–1.5	0.9	1	0.6–1.6	0.9
Socioeconomic rank *n* (%)	Low	25 (12.6)	25 (4.3)	1		Ref.	1		
	Intermediate	136 (68.3)	393 (68)	0.4	0.2–0.7	0.0	0.3	0.2–0.6	0.0
	High	38 (19.1)	160 (27.7)	0.3	0.1–0.5	<0.001	0.2	0.1–0.6	0.0
Health Maintenance Organization *n* (%)	Clalit Health services	80 (38.8)	253 (43.0)	1		Ref.	1		
	Maccabi Healthcare services	55 (26.7)	151 (25.7)	1.1	0.7–1.7	0.5	1.4	0.8–2.2	0.2
	Le’umit	26 (12.6)	52 (8.8)	1.6	0.9–2.7	0.1	1.8	0.9–3.2	0.0
	Me’uhedet	45 (21.8)	132 (22.4)	1.1	0.7–1.6	0.7	1.1	0.7–1.9	0.6
Reason to screening *n* (%)	Routine F/U	155 (79.5)	494 (92.0)	1		<0.0001	1		
	Risk factor	40 (20.5)	43 (8.0)	2.9	1.8–4.7		3.0	1.8–4.9	<0.001
Religion *n* (%)	Arab	27 (12.9)	38 (6.4)	1		0.0	1		
	Jewish & others	182 (87.1)	557 (93.6)	0.4	0.3–0.8		1.3	0.6–2.8	0.5

## Discussion

The incidence of invasive EOGBS disease among newborns in Israel is low, and consists of a multiyear average of 0.26 per 1000 live births (for the years 2014–2010). The low rate directed the decision to adopt a risk-based approach for the prevention of EOGBS.

Our survey demonstrated that despite the lack of universal GBS screening policy in Israel, about one third of the women surveyed were tested for GBS carriage during pregnancy. Most of these women had the test as part of routine pre-natal care or as a result of their request, particularly, in those women who underwent the testing on week 35 or later of preganacy. Furthermore, about 40 % of the women were not tested during the recommended time for screening, but rather before week 35 or after week 37 of pregnancy. The relatively high rate of GBS testing reported by women with no known risk factors for GBS carriage, suggests that the test is performed in Israel in a substantial number of cases despite the lack of recommendation for a universal GBS screening. This may be due to familiarity of physicians with the universal screening practiced in other countries, and concern of law suits. Our findings that GBS testing was more frequent in women belonging to the Jerusalem district and among members of certain HMOs, suggest that GBS testing practices may vary due to differences in the practices of physicians working for a specific HMO and in specific locations. Variability could also exist in the practice of different doctors belonging to the same HMO and district; however, additional research is required to address this issue.

The finding that women who underwent GBS testing were more likely to be of a higher socioeconomic status may reflect their awareness of universal screening performed elsewhere in the world. Differences in knowledge about the GBS colonization status by women giving birth, was previously described. A retrospective study from California, USA, demonstrated that prior to the implementation of universal GBS screening, black women had a lower probability of having GBS carriage information available at time of delivery [11].

Although age was found to be associated with undergoing GBS testing, the small age difference between those women who underwent GBS testing and those who did not, was minimal (Table 3).

Our study showed that lower socioeconomic status and having a medical reason for GBS testing were associated with GBS colonization. Lower socioeconomic status was also associated with lower GBS testing rates among women in our survey. Therefore, our study suggests that women who are least likely to be colonized with GBS undergo the test most frequently, a situation that may be associated with unnecessary monetary expenses.

Our finding that GBS carriers were more likely to belong to a low socioeconomic rank is interesting. Other reports addressing the association between GBS colonization and socioeconomic status showed mixed results. A study from the USA showed lower rates of GBS carriage among more educated women [12], and a study from Mexico demonstrated higher rates of GBS colonization among women residing in poor areas and of low socioeconomic status [13]. On the other hand, in a study from Turkey, GBS carriage was found more frequently among women of middle socioeconomic status [14]. Other studies did not find an association between GBS colonization and socioeconomic status [15, 16].

We found that when GBS testing was done, it was performed according to the recommended timing (35 to 37 weeks) only in 58 % of cases. Although these recommendation were made to capture most women prior to delivery, performing the test prior to week 35 is the most problematic. Although in our study, 22 % of the women undergoing the test prior to week 35, reported a valid reason for doing so (rupture of membranes or contractions with marked cervical changes prior to week 37), the majority did not. A study by Yancey et al. demonstrated that performing the test six weeks or more prior to delivery, decreases significantly its sensitivity, specificity, as well as positive and negative predictive values [17]. In our study 15 % of women performing GBS testing, underwent the test prior to week 35. In a recent study from Tennessee, where universal GBS screening is practiced, 26 % of pregnant women underwent GBS testing prior to week 35 [18]. These findings suggest that in a significant proportion of women undergoing GBS testing, the test is performed too early, regardless of the approach used for preventing EOGBS. Thus, their results may be irrelevant for decision-making during labor.

The major strength of our study was its nationwide scope, making it representative of the national situation. It was carried out in 29 hospitals out of the existing 30 general hospitals in Israel. Only one small peripheral hospital was excluded from the survey. However, due to the low number of births in this hospital (2 per day on average), it is anticipated that its inclusion in our study would have added approximately 46 subjects, which would have constituted only 1.5 % of the study population, and thus would not have had a significant impact on our results.

This assessment was done only during one month of the year. Though no seasonality is known for GBS, gathering data during a longer period may have altered some of the results. In addition, only 50 % of the women who underwent the test had documentation at the time of delivery. Another limitation of this study is self-report by women as to whether they were screened and what their result was.

Currently, it is unknown how many women undergo GBS screening during pregnancy in other countries that use risk-based approach. Based on our results, we believe that it is important to conduct similar studies in those countries, in order to assess, on one hand, compliance with health policies, and on the other hand, weather the existing health policies require adjustments.

The continually low rates of morbidity due to GBS in Israel during years 2006–2014 [8], and the fact that most women who were tested for GBS carriage did not have medical reasons warranting testing, suggest that the current policy of giving intrapartum antibiotic prophylaxis (ISAP) during labor and delivery based on the presence of risk factors, rather than based on the results of GBS testing at weeks 35–37, is appropriate for Israel. The current practice of performing GBS screening during pregnancy, especially not during the recommended time-frame and in the absence of medical reasons, suggests an unnecessary utilization of resources. Although testing for GBS colonization is best performed during weeks 35 to 37 weeks of pregnancy, the presence or absence of GBS during that time-period does not always reflect colonization during labor and delivery [17]. Thus, it is important to maintain the risk factor-based approach for preventing Early-Onset GBS disease in newborns.

## Conclusions

Our research proved that approximately a third of the expectant mothers in our survey have undergone GBS testing during pregnancy, and that the test was performed in those populations least at risk for GBS carriage. Furthermore, in over 40 % of the cases the test was not done at the recommended timing. Based on these findings, it appears that most GBS carriers are not detected. Thus, the performance of GBS testing during pregnancy cannot explain the low rate of EOGBS in Israel.

## References

[1] Homer CSE, Scarf V, Catling C, Davis D (2014). Culture-Based Versus Risk-Based Screening for the Prevention of Group B Streptococcal Disease in Newborns: A Review of National Guidelines. Women Birth.

[2] Melin P, Efstratiou A (2013). Group B Streptococcal Epidemiology and Vaccine Needs in Developed Countries. Vaccine.

[3] Verani JR, McGee L, Schrag SJ, Prevention of Perinatal Group B Streptococcal Disease. Revised Guidelines from CDC. Department of Health and Human Services, Centers for Disease Control and Prevention. MMWR. 2010;59(No. RR-10):1–32. 21088663

[4] Law MR, Palomaki G, Alfirevic Z, Gilbert R, Heath P, McCartney C, Reid T, Schrag S (2005). The Prevention of Neonatal Group B Streptococcal Disease: A Report by a Working Group of the Medical Screening Society. J Med Screen.

[5] Money D, Allen VM, Yudin MH, Toronto ON, Bouchard C, Boucher M, Caddy S, Castillo E, Murphy KE, Ogilvie G (2013). The Prevention of Early-Onset Neonatal Group B Streptococcal Disease. J Obstet Gynaecol Can.

[6] Berardi A, Di Fazzio G, Gavioli S, Di Grande E, Groppi A, Papa I, Piccinini G, Simoni A, Tridapalli E, Volta A (2011). Universal Antenatal Screening for Group B Streptococcus in Emilia-Romagna. J Med Screen.

[7] Israel ministry of health. Guideline for group B streptococcus screening among pregnant women [Hebrew]. 2005. pp. 1–3.

[8] Israel center for disease control, Israel ministry of health. Incidence of Early-Onset Neonatal Invasive Group B Streptococcal Disease in Israel, 2014–2010. 2015. [Hebrew].

[9] Israel Society of Obstetrics and Gynecology. Position Document No.22. 2011. pp. 4–26.

[10] Central Bureau of Statistics. Characterization and Classification of Local Authorities by the Socio-Economic Level of the Population. 2006. http://www.cbs.gov.il/www/publications/local_authorities06/local_authorities_e.htm. Accessed 7 Sept 2016.

[11] Bryant AS, Cheng YW, Caughey AB (2011). Equality in Obstetrical Care: Racial/Ethnic Variation in Group B Streptococcus Screening. Matern Child Health J.

[12] Regen JA, Klebanoff MA, Nugent RP (1991). The Epidemiology of Group B Streptococcal Colonization in Pregnancy. Obstet Gynecol.

[13] Cordero-Ocampo B. Factores Asociados a La Colonización Por Streptococcus Del Grupo B En Mujeres Embarazadas De Los Altos, Chiapas. Salud pública de México. 2000;5:413–21. 11125626

[14] Eren A, Kucukercan M, Oguzoglu N, Unal N, Karateke A (2005). The Carriage of Group B Streptococci in Turkish Pregnant Women and Its Transmission Rate in Newborns and Serotype Distribution. Turk J Pediatr.

[15] Hickman ME, Rench MA, Ferrieri P, Baker CJ (1999). Changing Epidemiology of Group B Streptococcal Colonization. Pediatrics.

[16] Papapetropoulou M, Kondakis XG (1987). A Study of Risk Factors of Vaginal Colonization with Group B Streptococci in Pregnancy. Eur J Epidemiol.

[17] Yancey MK, Schuchat A, Brown LK, Ventura VL, Markenson GR (1996). The Accuracy of Late Antenatal Screening Cultures in Predicting Genital Group B Streptococcal Colonization at Delivery. Obstet Gynecol.

[18] Goins WP, Talbot TR, William S, Edwards KM, Craig AS, Schrag SJ, Van Dyke MK, Griffin MR (2010). Adherence to Perinatal Group B Streptococcal Prevention Guidelines. Obstet Gynecol.

